# The Hospital World

**Published:** 1910-09-24

**Authors:** 


### The Hospital World.

### Elections and Appointments.

Mr. P. R. Wrigley, F.R.C.S. (Eng.), has been appointed an Honorary Assistant Surgeon at the Manchester Royal Infirmary.

Mr. G. P. L. Claridge, M.B., B.Sc.Lond., has been appointed honorary pathologist and bacteriologist to the Norfolk and Norwich Hospital.

### Personalia.

The King has granted his patronage to the London Fever Hospital.

Mrs. Mateo Clark, 182 Coleherne Court, S.W., has been appointed Lady President of the Clapham District (Parliamentary Division) of the League of Mercy.

Prince Francis of Teck, who has been so energetically busied in furthering the appeal on behalf of the Middlesex Hospital, is laid up for a few days in consequence of a nasal operation which he underwent last week. We understand that the Prince is making excellent progress, and expects soon to be able to resume his work in connection with the hospital.

At a special meeting of the General Committee of the Royal Southern Hospital, last week, four candidates, selected out of a large number of applicants for the position of matron, were interviewed. The vacancy has arisen through the retirement of Miss Mary Williams. After going fully into the merits of the four candidates, the Committee appointed Miss Lucy Eleanor Jolley to the position. Miss Jolley was trained at Guy's Hospital, London, where she received rapid promotion. In addition to holding the Guy's medal, she also holds the Guy's certificate, and the certificates of the Central Midwives Board and the Incorporated Society of Trained Masseuses.

Mr. Barlow, a well-known figure at the Shirley Children's Hospital, Southampton, has now resigned his vice-presidency, much to everyone's regret. His enthusiastic support of the institution has been constant, as the Barlow fund, which has produced as much as £60 a year, has proved abundantly. Whether his mantle will fall as another vice-president, and his energy and the success of his fund be maintained, it is too early to say. It is hoped that his example will be followed, and his departure, together with that of Mr. J. MacLachlan, who has worked for twenty-six years as general surgeon, will be the signal for new enthusiasts to arise, that, though the workers come and go, the work may continue.

Rev. J. D. Hoysted, who has just resigned from the Billericay (Essex) Board of Guardians after more than forty years of personal service, started his career in the public charitable service after reading Charles Dickens' "Oliver Twist." This book created such an impression upon his mind that he decided to devote himself to the reform of the workhouse system so drastically pilloried by the novelist. At the last meeting of the Board which he attended Mr. Hoysted, in giving some of his reminiscences of the system at the time when he first became interested in it, dwelt upon the salutary changes that have been effected during the last quarter of a century. To-day admission to the infirmary is sought as a privilege, and many of the workhouse infirmaries are models of what such institutions should be. The improvement is largely due to such earnest-minded men as Mr. Hoysted, who have not spared time or trouble to interest themselves in the work, and whose career should prove an inspiring example to many others.

Mr. Shrewsbury Smith has been elected as vice-chairman of the Worcester General Infirmary in the place of the late Canon Littleton Wheeler.

Miss Stevenson, matron of the Bromhead Institute for Nurses at Lincoln, has been selected by the authorities of the London Hospital to undertake the responsible position of superintendent matron of a group of medical college hospitals in India. Miss Stevenson will take over her duties in November next, and will be accompanied by three sisters from the London Hospital, who will assist her in her work.

The Rev. Canon Robinson, who has held the office of chaplain to the York County Hospital since 1900, has sent in his resignation, to everyone's regret. Canon Robinson has taken this course only because he is leaving York, and the Rev. F. L. Perkins, rector of St. Maurice, has been appointed to succeed him. The delicate and important work of hospital chaplain is accorded its due but rarely nowadays; yet probably no one is more grateful than chaplains themselves that the unobtrusiveness of their work is respected. No other appointment in an institution perhaps depends for its value so much on the personal character of the man who holds it. We may be sure, then, that when a chaplain wins the esteem of his fellow-workers, that esteem is deserved. Canon Robinson takes with him everyone's good wishes.

